# The Effect of Combining Natural Terpenes and Antituberculous Agents against Reference and Clinical *Mycobacterium tuberculosis* Strains

**DOI:** 10.3390/molecules23010176

**Published:** 2018-01-15

**Authors:** Elwira Sieniawska, Rafal Sawicki, Marta Swatko-Ossor, Agnieszka Napiorkowska, Agata Przekora, Grazyna Ginalska, Ewa Augustynowicz-Kopec

**Affiliations:** 1 Department of Pharmacognosy with Medicinal Plant Unit, Medical University of Lublin, 20-093 Lublin, Poland; 2 Department of Biochemistry and Biotechnology, Medical University of Lublin, 20-093 Lublin, Poland; rafal.sawicki@umlub.pl (R.S.); martaswatkoossor@umlub.pl (M.S.-O.); agata.przekora@umlub.pl (A.P.); grazynaginalska@umlub.pl (G.G.); 3 Department of Microbiology, National Tuberculosis and Lung Diseases Research Institute, 01-138 Warsaw, Poland; a.napiorkowska@igichp.edu.pl (A.N.); e.kopec@igichp.edu.pl (E.A.-K.)

**Keywords:** FICI, MDR-TB, clinical isolates, natural terpenes, bisabolol, (*R*)-limonene

## Abstract

*Background*: On account of emergence of multi- and extensively drug-resistant *Mycobacterium tuberculosis* (Mtb) strains, combinations of drugs with natural compounds were tested to search for antibiotic activity enhancers. In this work we studied terpenes (α-pinene, bisabolol, β-elemene, (*R*)-limonene, (*S*)-limonene, myrcene, sabinene), which are the main constituents of essential oil obtained from *Mutellina purpurea* L., a plant with described antitubercular activity, to investigate their interactions with antibiotics against reference Mtb strains and multidrug-resistant clinical isolates. *Methods*: The serial dilution method was used to evaluate the minimal inhibitory concentration (MIC) of tested compounds, while the fractional inhibitory concentration index (FICI) was calculated for characterization of interactions. Moreover, IC_50_ values of tested compounds were determined using monkey kidney epithelial cell line (GMK). *Results*: The combinations of all studied terpenes with ethambutol or rifampicin resulted in a synergistic interaction. Bisabolol and (*R*)-limonene decreased the MIC for rifampicin at least two-fold for all tested strains, however no synergistic action was observed against virulent strains. The tested terpenes showed slight (bisabolol) or no cytotoxic effect against normal eukaryotic cells in vitro. *Conclusions*: The obtained enhanced activity (FICI < 0.5) of ethambutol and rifampicin against H37Ra strain under the influence of the studied terpenes may be correlated to the capability of essential oil constituents to modify bacterial resistance mechanisms in general. The observed differences in avirulent and virulent bacteria susceptibility to terpenes tested separately and in combinations with antibiotics can be correlated with the differences in the cell wall structure between H37Ra mutant and all virulent strains.

## 1. Introduction

Tuberculosis is still a global threat [1], but the emergence of multi- and extremely-drug resistant *Mycobacterium tuberculosis* (Mtb) strains makes the treatment of this disease much more difficult. Since circulating strains are resistant to various combinations of drugs and the development of new drug leads takes a long time, a different approach is to find adjuvants capable of enhancing the efficacy of antibiotics. An enhancement of microbes’ susceptibility to antibiotics was observed under the influence of natural products [2], especially, essential oils [3,4,5,6,7]. Essential oils are known for their positive antimicrobial action and many possible mechanisms of their activity against a wide range of bacterial strains have been proposed [8,9,10,11]. The studies on synergy between essential oils components [12] as well as on the synergy between essential oils and antibiotics against wide range of microorganisms [13] indicates the possible impact of these compounds on restoring the sensitivity of resistant bacteria to antibiotics. Positive, synergistic interactions (FICI ≤ 0.5) were previously described for the antibiotics ciprofloxacin, gentamicin and polymyxin B with *Eucalyptus camaldulensis* essential oil against multidrug-resistant *Acinetobacter baumannii* isolates [3], for norfloxacin and *Pelargonium graveolens* essential oil against *Bacillus cereus* and *Staphylococcus aureus* [14], for ticarcillin, imipenem, gentamicin and tobramycin and *Origanum compactum* essential oil against *Pseudomonas putida* [15], to name just a few. However, very little is known about the influence of combinations of antibiotics and essential oils constituents against Mtb. Only *trans*-cinnamic acid with amikacin as well as *cis*-cinnamic acid with rifampicin were mentioned to have positive action against isolates of multi-drug resistant *M. tuberculosis* [5,6]. For this reason we selected several natural terpenes (α-pinene, bisabolol, β-elemene, (*R*)-limonene, (*S*)-limonene, myrcene, sabinene), which are the main constituents of the essential oil obtained from *Mutellina purpurea* L. (syn. *Ligusticum mutellina*), a plant with described antitubercular activity [16], to investigate their interactions with antibiotics against different mycobacterial strains. A monoterpene α-pinene is the most frequently found in Nature [17]. It is a main constituent in essential oils obtained from coniferous trees and shrubs [18,19], however it was also described in many other essential oils like *Rosmarinus officinalis*, *Satureja montana* [20,21], *Eucalyptus globulus* [22], *Melaleuca leucadendron*, [23], many other herbs and some citrus fruits essential oils. *Myoporum crassifolium* and *Matricaria recutita* are the most abundant sources of bisabolol, although it is present in many plants such as lavender or rosemary [17,24]. Rhizome of curcuma is used for β-elemene isolation, however this compound can be obtained also from *Michelia figo*, *Alisma orientale*, *Solidago decurrens* or *Nigella damascena* essential oils [25]. Limonene is known from citrus essential oils, where it accounts up to 90%, and is present in both enantiomeric forms ((−)-(*S*)-limonene and (+)-(*R*)-limonene) in many other essential oils [17]. Myrcene is other compound very widespread in Nature. It was described in most of the common highly fragrant herbs and spices, among which *Humulus lupulus*, *Laurus nobilis*, *Thymus vulgaris*, *Cymbopogon schoenanthus* or *Ocimum basilicum* contain high levels of this compound [17]. Sabinene is frequently present in essential oils. It is a major constituent in *Juniperus scopulorum* essential oil [26], it is also detected is higher amounts in *Myristica fragrans* [27], *Piper nigrum* [28] or *Daucus carota* essential oils [29].

The previous studies showed that α-pinene, myrcene, limonene, bisabolol and *M. purpurea* essential oilalter the mycobacterial cell shape and homogeneity [30], thereby they may enhance the action of antibiotics. Also the positive influence of these terpenes on activity of first line antibiotics against isolated Mtb was observed [31]. Hence, in this work we aimed to investigateif natural terpenes show any synergistic action with antituberculous agents against multi-drug resistant MTb clinical strains.

## 2. Results

### 2.1. Spoligotyping

The spoligotyping is an analysis of polymorphism in direct repeat region in *M. tuberculosis* DNA, which is very diverse in different clinical isolates. It can provide a genotypic classification of investigated strains [32]. The spoligotyping of isolated strains enabled us to the assign genotypic lineages according to SITVIT2 Database. Three strain families: ill-defined T family (clades SIT53 and SIT1558), Beijing and Haarlem family (sublineage H1) were identified (Table 1). The clade assignment demonstrated that susceptible strain belonged to T1 53 spoligotyp, while drug resistant strains were classified as T1 1558, H1 47 and Beijing 1 (Table 1).

### 2.2. Reference Avirulent Strain H37Ra

The susceptibility testing for compounds investigated separately revealed that growth of H37Ra strain was inhibited by the lowest concentrations of terpenes compared to other strains. The most effective antimycobacterial activity was shown by β-elemene and the limonenes (32 μg/mL) (Table 2). The evaluation of tuberculostatic activity of antibiotics in the presence of subinhibitory concentrations of terpenes showed that all tested compounds enhanced the activity of ethambutol and rifampicin against H37Ra strain. Several terpenes potentiated also the action of isoniazid (Table 3). When terpenes were tested in the presence of sub-inhibitory concentrations of antibiotics, the activity of terpenes was potentiated and MIC values obtained for terpenes were decreased several dilutions in different combinations (Table 4). The calculation of fractional inhibitory concentration indices enabled to find a synergistic action between antibiotics (ethambutol and rifampicin) and terpenes (Table 5).

### 2.3. Virulent, Sensitive and Drug-Resistant Strains

Among tested terpenes, bisabolol showed the highest activity against all investigated strains, sensitive and resistant as well. The lowest MIC value obtained for this compound on H37Rv and 192 strains equaled to 16 μg/mL. Likewise, INH-resistant strain 126, was quite susceptible to bisabolol, compared to the other terpenes (Table 2). The antimycobaterial activity of (*R*)-limonene followed bisabolol and was rather similar against all tested strains (MIC = 128–256 μg/mL). The weakest activity was observed for other terpenes against multidrug resistant strains (MIC ≥ 512 μg/mL) (Table 2). When antibiotics were tested in a presence of terpenes, bisabolol and (*R*)-limonene positively influenced the activity of antibiotics against majority of investigated strains. MIC values obtained for rifampicin was lowered at least two-fold in the presence of bisabolol and (*R*)-limonene for drug resistant isolates and reference strains. Similar pattern was observed for ethambutol, however the multidrug resistant 85/13 strain was not influenced. In case of isoniazid, bisabolol and (*R*)-limonene decreased MIC values two fold for all drug resistant isolates (Table 3—bolded values). It is also worth to notice that antimycobacterial activity of antibiotics against susceptible isolate 192 was not influenced by any of tested compounds. The evaluation of tuberculostatic activity of terpenes in the presence of subinhibitory concentrations of antibiotics resulted in MIC values obtained for sabinene, myrcene and (*S*)-limonene equal to these obtained for individual compounds for all virulent strains (Table 4). Only slightly increased antimycobacterial action was observed for almost all strains and drug combinations. The FICI indices obtained for combinations of tested compounds indicated an indifference in case of all virulent strains (FICI > 0.5 but ≤4) (Table 5).

### 2.4. Cytotoxicity Evaluation

The cytotoxicity evaluation revealed that limonenes and α-pinene are non-toxic, as GMK cells (monkey kidney epithelial cells) maintained high viability (approx. 90%) upon exposure to the highest tested concentration (500 μg/mL). Thus, determination of IC_50_ values for these compounds was impossible because it appears to be much higher than 500 μg/mL (the highest tested concentration). Lower IC_50_ value was obtained for sabinene (348 μg/mL), whereas α-bisabolol showed slight cytotoxic effect against GMK cells (71.12 μg/mL) (Table 6). However, IC_50_ value of sabinene was still higher than IC_50_ value of reference drug—rifampicin (141.5 μg/mL). According to da Silva et al., myrcene also did not present cytotoxic activity up to a concentration of 200 μg/mL against a Vero cell line (another monkey kidney epithelial cell line) [33]. β-Elemene is used as a broad spectrum antineoplastic agent and has been the subject of many clinical trials [34], hence it is regarded as safe.

## 3. Discussion

A synergistic action of drug-drug combinations is defined as greater activity than the activity of the sum of the individual components [12]. This effect is desirable and leading to a better antimicrobial therapy efficacy. On account of emergence of multi- and extensively drug-resistant Mtb strains the combinations of drugs with natural compounds are tested to search for enhancers of antibiotics activity [35]. Also our study aimed to investigate the possible interactions between terpenes, constituents of essential oils, and antimycobacterial antibiotics against drug-resistant clinical isolates and reference Mtb strains. We also performed cytotoxicity evaluation of tested compounds against eukaryotic cells.

The in-vitro cytotoxicity determination for terpenes (α-bisabolol, (*S*)-limonene, (*R*)-limonene, α-pinene and sabinene) and literature data (myrcene, β-elemene) [33,34] showed that they are non-toxic against eukaryotic cells (green monkey kidney cells: Vero or GMK cell line).

The antimycobacterial activity of tested terpenes was described for a panel of Mtb strains (including clinical isolates) for a first time. However several of these compounds (sabinene, myrcene and limonene) were previously screened against reference virulent strain H37Rv. The MIC values obtained in our study for sabinene, myrcene and limonene were significantly higher than previously described by Andrade-Ochoa et al. [36] but this can be explained by different method applied for evaluation of inhibitory activity of tested compounds (visual evaluation, 21 day of incubation in our study and Alamar Blue technique, 8 days of incubation in the cited paper). Regardless the method used the activity of these three compounds was on the similar level. Among other terpenes, the interesting activity was shown by thymol and carvacrol (0.78 and 2.02 μg/mL, respectively), while the highest MIC values were obtained for *p*-cymene and β-caryophyllene (91 and 100 μg/mL, respectively) [36].

Taking into account the results of antimycobacterial activity of tested terpenes against different mycobacteria strains, it is difficult to correlate their activity with the structure, because the MIC values varied even by several orders for different strains, and the activity could not be described by any particular pattern. The quantitative structure–activity relationship studies performed by Andrade-Ochoa and coworkers revealed that the number of conjugated carbons, the number of phenolic and hydroxyl groups and the number of acceptor atoms of hydrogen bonds are the most important structural descriptors in the antimycobacterial activity of terpenes [36]. Compounds tested in this study were simple terpenes thus phenolic and hydroxyl groups nor oxygen atoms did not contribute to their antimycobacterial activity. The lowest MIC values observed for two-compounds combinations were descried for monocyclic sesquiterpene—bisabolol having a long side chain and for monocyclic monoterpene—(*R*)-limonene—with methyl and propylene groups. What is more interesting, there were significant differences in MIC values obtained for enantiomeric forms of limonene against mycobacterial strains (with exception of the avirulent strain H37Ra) and for combinations of limonenes with antiobiotics. (*R*)-Limonene occurs commonly in many plants but is especially abundant in citrus fruits, it has citrus odor, while the less often found (*S*)-limonene is a precursor in the biosynthesis of (−)-menthol and its odor is pine-like [37]. The distinction between these compounds is a different spatial arrangement of the propylene and methyl groups and it seems that more often naturally occurring isomer has also better antimicrobial properties, probably via specific interactions with mycobacterial cell envelope

The results from antimycobacterial assay showed that enhanced activity of ethambutol and rifampicin against H37Ra strain under the influence of studied terpenes may be correlated to the capability of essential oils constituents to modify the bacteria resistance mechanisms in general. Three possible scenarios can take place: inhibition of multidrug efflux pumps (farnesol in *Mycobacterium smegmatis*) [38], cell wall and membrane disturbance (β-elemene and R-limonene in *M. tuberculosis*) [39] and alterations of quorum-sensing (Rose, geranium, lavender, clove essential oils in *Escherichia coli*) [40]. These may lead to better influx and retention of antibiotics inside the cells resulting their better efficacy. Our previous observations have shown that studied terpenes cause the changes in mycobacterium H37Ra cells shape, cell wall thickness and cytoplasm homogeneity in terms of uniformity and consistency [30]. Bacteria exposed to terpenes became filamentous (continuing to elongate but not to divide) what usually occurs under the oxidative stress, nutrient limitation or DNA damage and alters DNA replication and cell division [41]. Also other authors described similar morphological changes in pathogenic and spoilage-forming bacteria cells structures. The loose of regular cell shape and changes in cytoplasm and membrane integrity were observed under the influence of tea tree oil and some terpenes [40,42,43]. These findings suggest that cell wall and membrane disturbances caused by essential oils constituents may mainly contribute to the better penetration of antibiotics into the cells and their enhanced activity when tested in combinations. 

However the important observations from this study are differences in avirulent and virulent bacteria susceptibility to terpenes tested individualy and in combinations with antibiotics. A comprehensive description of possible genetic variations, like multiple mutations, between virulent strain H37Rv and avirulent one, was described by Zheng and coworkers [44]. The changes found in H37Ra may account for its attenuation of virulence and various other phenotypic changes, like changes in bacteria susceptibility to antibiotics [44].Since synergistic interaction between antibiotics (ethambutol/rifmapicin) and terpenes was described only against avirulent strain H37Ra, the higher susceptibility of this strain to tested compounds may be related to the mutation in phoP gene, which is one of the factors leading to the inability of bacteria growth in human and murine macrophages [45,46,47]. The phoP gene encodes PhoP protein playing a key regulatory function in synthesis or transfer of metyl branched fatty acyl substituents found in polyketide-derived acyltrehaloses (sulfolipids SL, diacyltrehaloses DATs and polyacyltrehaloses PATs) [48,49]. The absence of these lipids in the cell wall was described in H37Ra mutant strain [47,50] and this may contribute to the better action of terpenes themselves and in combination with antibiotics. The antibacterial activity of terpenes leading to the increased membrane permeability is dependent on the bacterial membrane net surface charge [9], which in physiological conditions is negative [9,51,52,53]. However during interaction of bacteria with essential oils constituents it becomes less negative [9]. In case of H37Ra mutant strain, the lack of sulfolipids in cell wall may be responsible for the enhanced antibacterial activity since sulfation gives an anionic form to a complex lipids of mycobacterial envelope and improves the lipid solubilization [54]. What is more, the experiments performed by Trombetta et al., suggest a real transfer of terpenes through cell membrane and possibility of their interaction with intracellular elements [11] but this action is strongly dependent on the lipid composition of the cell envelope [9,11,55,56]. Hence the observed lack of synergistic action of combinations of terpenes and antibiotics in case of all virulent strains might be a consequence of their richer composition of cell envelope [11], especially regarding the presence of midchain methyl-branched fatty acids (10-methylhexadecanoic(16:1me(10)), 10-methyloctadecanoic acid (18:1me(10)), and 10-methylnonadecanoic acid (19:1me(10)) which are associated with tolerance mechanisms to xenobiotics and with the maintenance of membrane fluidity under different stress conditions [57,58].

The literature data show synergistic interactions among antibiotics: isoniazid, rifampicin, and ethambutol (18.1% of combinations with FICI = 0.6) or ofloxacin, rifampicin, and ethambutol (91.3% of combinations with FICI = 0.31–0.62) against *M. tuberculosis* H37Rv [59] or between antibiotics and natural products: oleic acid (in combination with isoniazid, rifampicin and ethambutol, FICI in a range 0.09–0.36), 7-methyljuglone (in combination with isoniazid, FICI = 0.2 and with rifampicin, FICI = 0.5), usnic acid (with rifampicin, FICI = 0.25–0.38) against *M. tuberculosis* H37Rv strain and some Mtb drug-resistant clinical isolates [2,60,61], however little is known about their mechanism of action. Apart from positive results, the lack of synergy against different bacterial species was also documented. In the study performed by Rey-Jurado et al., the indifference (FICI = 1.5–3) was observed for all three-drug combinations of second-choice antituberculous antibiotics tested against multidrug-resistant Mtb isolates, showing all these combinations to be equally effective [62]. Also the combination of carvacrol, eugenol and cinnamaldehyde encapsulated within lipid nanocapsules and doxycycline tested against *Acinetobacter baumannii* SAN, *A. baumannii* RCH, *Klebsiella pneumoniae*, *Escherichia coli* and *Pseudomonas aeruginosa* resulted in indifference (FICI = 0.7–1.30) [63]. Nevertheless, despite the lack of synergy in these species, the scanning electron microscopy images showed holes in bacterial envelope and leakage of cellular contents after exposure to tested combination [63] indicating their influence on the bacterial cell wall/membrane which probably can be attributed to the action of essential oil constituents. What is more, we also observed that MIC values obtained for rifampicin was lowered at least two-fold in the presence of bisabolol and (*R*)-limonene for drug resistant isolates and reference strains although the synergy in action wasn’t observed for these strains. This observation may suggest that disturbances in a cell envelope integrity may facilitate the intake of antibiotic also in virulent strains, however the extent of changes was too low to produce synergy. The other interesting observation from our study and from literature data [60,61] showed that rifampicin produce positive interactions with natural compounds more often than INH, EMB or SM. This may be correlated with different target points of rifampicin (lipophilic compound inhibiting DNA-dependent RNA polymerase) [64] and terpenes (influencing cell wall and membrane), however it was not explained yet. Additional studies are needed for better understanding of interactions taking place in bacteria when antibiotics are combined with essential oils or its constituents.

## 4. Materials and Methods

### 4.1. Tested Compounds

The first line antibiotics: rifampicin, isoniazid and ethambutol as well as natural terpenes: α-pinene 98%; (*R*)-limonene 97%; (*S*)-limonene 98%; myrcene 90%, bisabolol 93% were purchased from Sigma-Aldrich (St. Louis, MO, USA). Sabinene 99% and β-elemene 97% were isolated. Separation of sabinene and β-elemene was performed on Spectrum high-performance counter-current chromatographic equipment (Dynamic Extraction Co., Ltd., Slough, Berkshire, UK). Sabinene was obtained from commercially available *Daucus carota* seeds essential oil, as described previously [29], while β-elemene was isolated from *Nigella damascena* seed essential oil [65]. Briefly, mixture of *n*-hexane, acetonitrile and *tert*-butyl-methyl ether in a ratio 2:1:0.1 (*v*/*v*) was used as two phase solvent system for sabinene purification, while β-elemene was separated with a mixture of petroleum ether, acetonitrile and acetone in a ratio of 2:1.5:0.5 (*v*/*v*). Both separations were performed in reversed phase mode with a mobile phase flow rate equal to 6 mL/min and the eluate was monitored at 210 nm. The obtained one-minute fractions, collected from the beginning of the run, were checked for content and purity by means of gas chromatography-mass spectrometry [29]. 200 and 280 mg of essential oil was used for semipreparative separation of β-elemene and sabinene, respectively.

### 4.2. Mycobacterial Strains

Two reference strains (avirulent Mtb H37Ra ATTC 25177 and virulent H37R_V_ ATTC 25618) as well as five clinical isolates (192—strain susceptible; 12331—strain resistant to isoniazid (INH), rifampicin (RMP) and ethambutol (EMB); 256/16—strain resistant to RMP; 85/13—strain belonging to a Beijing family, resistant to streptomycin, INH, RMP and EMB; 126—strain resistant to INH) were investigated. Clinical isolates were collected in National Tuberculosis and Lung Diseases Research Institute (Warsaw, Poland). Drug susceptibility of isolates to first-line antibiotics (EMB, streptomycin—SM, INH and RMP) was verified using a conventional indirect proportion method on Löwenstein-Jensen (L-J) medium [66].

### 4.3. Spoligotyping

All isolates were analyzed by spoligotyping as described previously [67]. In brief, amplified biotinylated PCR products were hybridized to a set of 43 oligonucleotides covalently bound to a membrane (Isogen Life Science B.V., Utrecht, The Netherlands). The resulting hybridization signals were revealed by enhanced chemiluminescence detection system (Amersham, Little Chalfont, UK) and were visualized by exposure to X-ray film (Hyperfilm ECL; Amersham) according to the manufacturer’s instructions. The profiles were obtained by photochemical development of X-ray film. The resulting spoligotypes were converted from binary format to an octal code for comparison with the SITVIT2 proprietary database of the Pasteur Institute of Guadeloupe database (available online at http://www.pasteur-guadeloupe.fr:8081/SITVITDemo). Major phylogenetic clades were assigned according to signatures provided in SpolDB4 [68].

### 4.4. Minimal Inhibitory Concentration Determination

Mtb strains were grown in Lowenstein-Jensen medium slants [69]. Colonies were suspended with sterile distilled water containing 5 mm glass beads and vortexed during 45 s. The supernatant was harvested and adjusted to 0.5 McFarland with a nephelometer (Becton, Dickinson & Company, Franklin Lakes, MD, USA). For minimal inhibitory concentration (MIC) testing the inoculum was transferred to Difco^TM^ Middlebrook 7H9 broth (Becton, Dickinson & Company, Franklin Lakes, MD, USA) supplemented sodium chloride, bovine albumin fraction V, dextrose and catalase (ADC enrichment, Becton, Dickinson & Company, Franklin Lakes, MD, USA) and incubated in a presence of tested substances or their combinations at 37 °C for 21 days. Afterwards, the lowest concentration at which no visible growth occurred was recorded to be the MIC value of the individual and combined antibacterial agents. The stock solutions of terpenes and antibiotics were prepared in dimethyl sulfoxide (DMSO). The individual MIC values for tested substances and antibiotics were determined in a range 0.125–512 µg/mL. The concentration of terpenes tested in a presence of sub-inhibitory concentration of antibiotics was in a range 0.5–512 µg/mL, whereas the range of concentration of antibiotics tested in a presence of sub-inhibitory concentration of terpenes was 0.0625–512 µg/mL for Mtb H37Ra and 0.125–512 µg/mL for other strains. The negative and positive control were consisted of medium without inoculum and medium inoculated with the same amount of bacteria as test vials kept in the same conditions as test vials, respectively. The highest concentration of DMSO used in the samples (0.5%) was tested to evaluate any possible bactericidal effect of the solvent. All experiments were performed according to the CLSI standards [70] and in triplicate.

### 4.5. Evaluation of Interactions between Terpenes and Antibiotics

The Fractional Inhibitory Concentration Index (FICI) was calculated as follows: FICI = FICA + FICB = A/MICA + B/MICB where A and B were the MIC of each tested compound in combination and MICA and MICB were the MIC of each tested compound individually. The obtained FICI values were then used to determine whether synergism (FICI ≤ 0.5), indifference (>0.5 FICI ≤ 4), or antagonism (FICI > 4) occurred between the tested agents [71].

### 4.6. Cytotoxicity Study

The cytotoxicity experiment was performed using African green monkey kidney epithelial cells (GMK) obtained from BIOMED-Lublin S.A. (Lublin, Poland). The cells were cultured in EMEM medium (ATCC-LGC Standards, Cumberland, ME, USA) supplemented with 10% foetal bovine serum (FBS, Pan-Biotech, Aidenbach, Germany), penicillin (100 U/mL), and streptomycin (100 μg/mL) (Sigma-Aldrich, St. Louis, MO, USA). The GMK cells were maintained at 37 °C in a humidified atmosphere of 5% CO_2_ and 95% air. The GMK cells were seeded in 96-multiwell plates in 100 μL of the complete EMEM medium at a concentration of 3 × 10^5^ cells/mL (3 × 10^4^ cells/well) and the plates were maintained at 37 °C for 24 h. Then, the culture medium was replaced with 100 µL of different concentrations of tested agents and 2 reference anti-mycobacterial drugs—rifampicin and isoniazid. The stock solutions of all compounds were prepared in DMSO (Sigma-Aldrich, St. Louis, MO, USA), thus the highest solvent concentrations (1% and 0.5% DMSO) used in the experiment were tested in parallel to exclude potential toxicity associated with the use of DMSO. Different concentrations of the tested agents were obtained by six serial 2-fold dilutions using culture medium (the highest tested concentration was equal 500 µg/mL). GMK cells maintained in culture medium without anti-mycobacterial agent served as a negative control of cytotoxicity. The cells were exposed to tested compounds and reference drugs for 48 h, then cytotoxicity test was performed using MTT assay (Sigma-Aldrich, St. Louis, MO, USA) as it was described previously [72]. The cytotoxicity test was repeated in three separate experiments (*n* = 3) and each experiment was carried out in quadruplicate. The IC_50_ values (concentration reducing viability of GMK cells by 50%) were determined using GraphPad Prism 5, Version 5.03 software (GraphPad Software, Inc., La Jolla, CA, USA). 

## 5. Conclusions

The performed study has shown positive synergistic results for combinations of natural terpenes and antimycobacterial antibiotics (INH, RMP) against avirulent H37Ra strain. The other observation were differences between avirulent and virulent bacterial susceptibility to terpenes tested alone and in combinations with antibiotics, which can be correlated with the differences in the cell wall structure between H37Ra mutant and all virulent strains. The presence of mid-chain methyl-branched fatty acids in the mycobacterial cell envelope resulted in better tolerance mechanisms to tested terpenes in virulent strains, however bisabolol and (*R*)-limonene also lowered the MIC values obtained for antibiotics in these less sensitive bacteria.

## Figures and Tables

**Table 1:** Characterization of investigates strains.

Strain	Resistance Pattern	Spoligotyp
H37RaATTC 25177	susceptible	H37Ra
H37RV ATTC 25618	susceptible	H37Rv
192	susceptible	T1 53
12331	IRE	H1 47
253/16	IRE	T1 1558
85/13	SIRE	Beijing 1
126	INH	T1 1558

IRE—isoniazid, rifampicin and ethambutol resistant; RMP—rifampicin; SIRE—resistant to streptomycin, INH, RMP and EMB; INH—isoniazid.

**Table 2:** The minimal inhibitory concentration values (MIC) obtained for compounds tested separately.

	MIC μg/mL						
	H37Ra	H37Rv	192	12331	253/16	85/13	126
EMB	4	0.25	<0.125	16	64	16	0.25
RMP	1	0.25	<0.125	256	4	128	0.25
INH	0.125	<0.125	<0.125	16	8	16	0.5
α-pinene	128	128	128	>512	256	512	128
sabinene	64	128	128	>512	256	512	128
bisabolol	64	16	16	256	128	128	32
β-elemene	32	256	256	256	>512	>512	256
myrcene	128	256	256	512	>512	>512	256
(*S*)-limonene	32	256	256	512	>512	>512	256
(*R*)-limonene	32	128	128	128	256	256	128

EMB—ethambutol; RMP—rifampicin; INH—isoniazid.

**Table 3:** The minimal inhibitory concentration values (MIC) obtained for antibiotics tested in the presence of subinhibitory concentrations of terpenes CLSI.

	**MIC (μg/mL) for EMB in a Presence of Terpenes**						
	**H37Ra**	**H37Rv**	**192**	**12331**	**253/16**	**85/13**	**126**
α-pinene	1	0.125	<0.125	16	32	16	0.25
sabinene	0.5	0.25	<0.125	16	64	16	0.25
bisabolol	1	0.125	<0.125	8	32	16	0.125
β-elemene	1	0.25	<0.125	16	64	16	0.25
myrcene	0.5	0.25	<0.125	16	64	16	0.25
(*S*)-limonene	0.5	0.25	<0.125	16	32	16	0.25
(*R*)-limonene	1	0.125	<0.125	8	32	16	0.125
	**MIC (μg/mL) for RMP in a Presence of Terpenes**						
	**H37Ra**	**H37Rv**	**192**	**12331**	**253/16**	**85/13**	**126**
α-pinene	0.0625	0.25	<0.125	256	4	128	0.25
sabinene	0.0625	0.25	<0.125	256	4	128	0.25
bisabolol	0.0625	0.125	<0.125	128	2	64	0.125
β-elemene	0.0625	0.25	<0.125	256	4	128	0.25
myrcene	0.125	0.25	<0.125	256	4	128	0.25
(*S*)-limonene	0.125	0.25	<0.125	256	4	128	0.25
(*R*)-limonene	0.125	0.125	<0.125	128	2	64	0.125
	**MIC (μg/mL) for INH in a Presence of Terpenes**						
	**H37Ra**	**H37Rv**	**192**	**12331**	**253/16**	**85/13**	**126**
α-pinene	0.125	<0.125	<0.125	16	8	16	0.5
sabinene	0.0625	<0.125	<0.125	16	8	16	0.5
bisabolol	0.125	<0.125	<0.125	8	4	8	0.25
β-elemene	0.0625	<0.125	<0.125	16	8	16	0.5
myrcene	0.0625	<0.125	<0.125	16	8	16	0.5
(*S*)-limonene	0.0625	<0.125	<0.125	16	8	16	0.5
(*R*)-limonene	0.125	<0.125	<0.125	8	4	8	0.25

EMB—ethambutol; RMP—rifampicin; INH—isoniazid.

**Table 4:** The minimal inhibitory concentration values (MIC) obtained for terpenes tested in the presence of subinhibitory concentrations of antibiotics.

	**MIC (μg/mL) for α-Pinene in the Presence of Antibiotics**						
	**H37Ra**	**H37Rv**	**192**	**12331**	**253/16**	**85/13**	**126**
EMB	8	128	128	>512	256	512	128
RMP	8	64	64	>512	256	512	64
INH	128	64	64	>512	256	512	128
	**MIC (μg/mL) for Sabinene in the Presence of Antibiotics**						
	**H37Ra**	**H37Rv**	**192**	**12331**	**253/16**	**85/13**	**126**
EMB	4	128	128	>512	256	512	128
RMP	4	128	128	>512	256	512	128
INH	8	128	128	>512	256	512	128
	**MIC (μg/mL) for Bisabolol in the Presence of Antibiotics**						
	**H37Ra**	**H37Rv**	**192**	**12331**	**253/16**	**85/13**	**126**
EMB	2	16	16	256	128	128	32
RMP	4	8	8	64	128	128	32
INH	32	8	16	64	128	128	16
	**MIC (μg/mL) for β-Elemene in the Presence of Antibiotics**						
	**H37Ra**	**H37Rv**	**192**	**12331**	**253/16**	**85/13**	**126**
EMB	2	128	128	256	256	>512	256
RMP	2	128	128	256	256	256	256
INH	4	128	128	256	256	>512	256
	**MIC (μg/mL) for Myrcene in the Presence of Antibiotics**						
	**H37Ra**	**H37Rv**	**192**	**12331**	**253/16**	**85/13**	**126**
EMB	8	256	256	512	>512	>512	256
RMP	2	256	256	512	>512	>512	256
INH	64	256	256	512	>512	>512	256
	**MIC (μg/mL) for (*S*)-Limonene in the Presence of Antibiotics**						
	**H37Ra**	**H37Rv**	**192**	**12331**	**253/16**	**85/13**	**126**
EMB	2	256	256	512	>512	>512	256
RMP	0.5	256	256	512	>512	>512	256
INH	32	256	256	512	>512	>512	256
	**MIC (μg/mL) for (*R*)-Limonene in the Presence of Antibiotics**						
	**H37Ra**	**H37Rv**	**192**	**12331**	**253/16**	**85/13**	**126**
EMB	2	64	64	128	128	256	128
RMP	0.5	64	64	64	256	256	256
INH	32	64	64	128	128	256	128

EMB—ethambutol; RMP—rifampicin; INH—isoniazid.

**Table 5:** The fractional inhibitory concentration indices obtained for combinations of terpenes and antibiotics.

	FICI Values Obtained for Mycobacterial Strains						
	H37Ra	H37Rv	192	12331	256/16	85/13	126
INH/bisabolol	2	1.5	2	0.75	1.5	1.5	1
INH/myrcene	1	2	2	2	2	2	2
INH/(*R*)-limonene	2	1.5	1.5	1.5	1	1.5	1.5
INH/(*S*)-limonene	0.6	2	2	2	2	2	2
INH/sabinene	0.6	2	2	2	2	2	2
INH/α-pinene	2	1.5	1.5	2	2	2	2
INH/β-elemene	0.6	1.5	1.5	2	1.5	2	2
EMB/bisabolol	1	1.5	2	1.5	1.5	2	1.5
EMB/myrcene	0.2	2	2	2	2	2	2
EMB/(*R*)-limonene	0.3	1	1.5	1.5	1	2	1.5
EMB/(*S*)-limonene	0.1	2	2	2	1.5	2	2
EMB/sabinene	0.2	2	2	2	2	2	2
EMB/α-pinene	0.3	1.5	2	2	1.5	2	2
EMB/β-elemene	0.3	1.5	1.5	2	1.5	2	2
RMP/bisabolol	0.1	1	1.5	0.75	1.5	1.5	1.5
RMP/myrcene	0.1	2	2	2	2	2	2
RMP/(*R*)-limonene	0.1	1	1.5	1	1.5	1.5	2.5
RMP/(*S*)-limonene	0.1	2	2	2	2	2	2
RMP/sabinene	0.1	2	2	2	2	2	2
RMP/α-pinene	0.1	1.5	1.5	2	2	2	1.5
RMP/β-elemene	0.1	1.5	1.5	2	1.5	1.5	2

Synergism ≤ 0.5; indifference >0.5 to ≤4; antagonism > 4 EMB—ethambutol; RMP—rifampicin; INH—isoniazid.

**Table 6:** Cytoxicity of tested terpenes and antibiotics.

Tested Agent	IC_50_ (μg/mL)
α-Bisabolol	71.12
(*S*)-Limonene	>500
(*R*)-Limonene	>500
α-Pinene	>500
Sabinene	348
β-Elemene	ND
Myrcene	200 *
Rifampicin	141.5
Isoniazid	>500

* determined on Vero cell Line [31]; ND—not determined.
